# Providing optimal care in the neonatal care units in India: How Covid-19 exacerbated existing barriers

**DOI:** 10.1371/journal.pgph.0000393

**Published:** 2024-05-02

**Authors:** Lisa Messersmith, Cherryl Kolhe, Alyana Ladha, Prabir Das, Sowmya R. Rao, Marym Mohammady, Emily Conant, Rejesh Bose, Nithya Ramanathan, Archana Patel, Patricia L. Hibberd

**Affiliations:** 1 Department of Global Health, Boston University School of Public Health, Boston, Massachusetts, United States of America; 2 Lata Medical Research Foundation, Nagpur, India; 3 Nexleaf Analytics, Los Angeles, California, United States of America; 4 Datta Meghe Institute of Medical Sciences, Wardha, India; Australian National University Medical School, AUSTRALIA

## Abstract

Nearly one quarter (600,000) of all neonatal deaths worldwide per year occur in India. To reduce neonatal mortality, the Indian Ministry of Health and Family Welfare established neonatal care units, including neonatal intensive care units and specialized neonatal care units to provide immediate care at birth, resuscitation for asphyxiation, postnatal care, follow up for high-risk newborns, immunization, and referral for additional or complex healthcare services. Despite these efforts, neonatal mortality remains high, and measures taken to reduce mortality have been severely challenged by multiple problems caused by the Covid-19 pandemic. In this qualitative study, we conducted seven focus group discussions with newborn care unit nurses and pediatric residents and 35 key informant interviews with pediatricians, residents, nurses, annual equipment maintenance contractors, equipment manufacturers, and Ministry personnel in the Vidarbha region of Maharashtra between December 2019 and November 2020. The goal of the study was to understand barriers and facilitators to providing optimal care to neonates, including the challenges imposed by the Covid-19 pandemic. Covid-19 exacerbated existing barriers to providing optimal care to neonates in these newborn care units. As a result of Covid-19, we found the units were even more short-staffed than usual, with trained pediatric nurses and essential equipment diverted from newborn care to attend to patients with Covid-19. Regular training of neonatal nursing staff was also disrupted due to Covid-19, leaving many staff without the skills to provide optimate care to neonates. Infection control was also exacerbated by Covid-19. This study highlights the barriers to providing optimal care for neonates were made even more challenging during Covid-19 because of the diversion of critically important neonatal equipment and staff trained to use that equipment to Covid-19 wards. The barriers at the individual, facility, and systems levels will remain challenging as the Covid-19 pandemic continues.

## Introduction

Nearly 2.6 million global neonatal deaths occur each year from predominantly preventable causes [1]. Of these 2.6 million deaths, approximately 25% (600,000 deaths) occur in India, where the proportion of neonatal deaths in children under age 5 years increased from 48% to 59% between 2000 and 2016 [1]. To reduce neonatal mortality, the Indian Ministry of Health and Family Welfare has focused on increasing the proportion of health facilities births and created and scaled up Special Neonatal Care Units (SNCUs) in public district and sub-district level hospitals [2] to provide Level II care [3], including immediate care at birth, resuscitation for asphyxiation, postnatal care, follow up for high-risk newborns, immunization, and referral for additional or complex healthcare services [4,5]. Trained neonatologists, staff nurses, and support staff provide care 24 hours a day in the SNCU.

Over the last 15 years, India has been encouraging pregnant women to deliver their babies in Institutions and the Public Health System has been strengthening facility based newborn care. This effort has resulted in a need for easily accessible newborn care outside tertiary care hospitals in major cities where most highly specialized Neonatal Intensive Care Units are usually housed. The Indian government specifically created Special Newborn Care Units (SCNUs) in district and sub-district level hospitals with more than 3,000 deliveries per year to provide care for newborns requiring more than basic levels of hospital care, but less than highly specialized care that is often difficult to access for babies born outside major cities. SCNUs (which have 12 or more beds) are separate units close to the labor and delivery units. Trained neonatologists, staff nurses and support staff provide care around the clock. While SNCUs have increased in numbers across India and now care for more than one million babies per year, there are challenges to reducing neonatal morbidity and mortality in these units, in part because there are no guidelines about the most cost effective and reliable equipment that should be available to achieve this goal.

Challenges to reducing neonatal morbidity and mortality in neonatal care units include the lack of continuing education for clinic staff and optimizing the use and functioning of medical equipment for monitoring at-risk newborns. A qualitative study assessing barriers and facilitators related to optimal obstetric and neonatal emergency care in primary care facilities in Bihar, India, reported three fourths of participants indicated the following barriers to providing adequate care for patients: lack of physical resources, shortage of providers, and a high patient load [6]. The same study reported the following facilitators: improved skills and confidence through training, increased number of trainings, mentorship systems for providers, and administrative guidance [6].

By the end of February 2023, India had reported nearly 44.7 million cases of Covid-19 and over 530,000 deaths, with five states accounting for the highest number of cases—Maharashtra, Kerala, Karnataka, Tamil Nadu and Andhra Pradesh [7]. Yet, the World Health Organization reported that cases and deaths due to Covid-19 were underreported across the globe, including in India [8], indicating that the Indian estimates also fall short of the actual cases and deaths due to Covid-19. With the rapid spread of Covid-19, demand for healthcare has only increased. Private healthcare remains expensive and unavailable to many households in India, leaving public healthcare as the only option [9]. The availability of public hospital beds remains at 0.55 beds per 1000 people at the country level and even lower at the state level for states such as Bihar, Jharkhand, Gujarat, Uttar Pradesh, Andhra Pradesh, Chhattisgarh, Madhya Pradesh, Haryana, Odisha, Assam, Manipur, and Maharashtra which comprise nearly 70% of India’s total population [9].

In response to Covid-19, India’s National Center for Disease Control outlined policy advice for each hospital area including the SNCU. Both adaptations of services and suspensions of services were recommended to reduce the spread of Covid-19, including conducting all routine SNCU follow-up via telehealth appointments (with the exception of sick newborns referred to SNCUs) and suspending all family participatory care within the SNCU [7,10].

This study elucidates the effect of Covid-19 on the neonatal care units, affecting care, staff training and administrative prioritization. It highlights the need for pandemic specific training, proper administrative communication and triage to better prepare for unforeseen circumstances without compromising on caring for neonates, that also form a vulnerable group of populations.

## Methods

### Design

In December 2019, prior to the Covid-19 pandemic, our team initiated a study on the barriers and facilitators to providing optimal care to neonates in NICUs and SNCUs with a focus on the current gaps and future needs with a focus on equipment. This focus was meant to inform future investments by the Indian government in appropriate equipment and equipment support for SNCUs. We conducted our research with this focus until the Covid-19 lockdown in March 2020 when data collection was suspended. Our team was able to restart data collection in July 2020 and began collecting additional data on the impact of Covid-19 on neonatal care. Our data collection ended in November 2020. This paper focuses on the impact of Covid-19 on providing optimal care for newborns in these units in one district in Maharashtra.

During our study in Maharashtra, all pregnant women were tested for Covid. If found to be positive they were transferred to the Covid care designated hospital, a separate building in the premises of the tertiary care Covid-designated hospital in Nagpur. Any woman presenting for delivery or any newborn found to be Covid positive was also referred to the tertiary care Covid-designated hospital in Nagpur.

Nine hospital facilities containing SNCUs and Neonatal Intensive Care Units (NICUs) were purposively selected for this study. Facilities included district general hospitals, sub-district hospitals, trust hospitals, and medical colleges. Focus group discussion (FGD) and key informant interview (KII) participants were licensed pediatricians, pediatric medical residents, medical officers or nurses who work in public or private tertiary care facilities with an operational SNCU or NICU, who consented to be interviewed and were over the age of 18. KII participants also included SNCU/NICU equipment distributors, annual maintenance contractors, equipment designers, and equipment manufacturers who worked in the study districts and were over the age of 18. Healthcare staff who did not work in or supervise the SNCU/NICU or who were not qualified as annual maintenance contractors, equipment designed or manufacturers under the age of 18 were excluded from the study. A total of 66 individuals (35 key informants and 31 FGD participants) participated in the study (please see Table 1 below for more detail). A total of seven FGDs (two with pediatric residents (3 and 4 participants) and five with SNCU/NICU staff nurses (4–6 participants in each) were conducted. One in-charge nurse at one facility refused to participate in a KII and did not give a reason. Two FGDs were planned for another facility, but these were not conducted due to a staff shortage caused by COVID. FGDs and KIIs explored perspectives on equipment function and use, gaps and needs for medical equipment for different types of newborn units, and the equipment-related barriers and facilitators to optimal care for neonates in these units.

**Table 1:** Participants in key informant interviews and focus groups by facility type.

Qualitative Data Collection by Facility Type							
		General Hospital* (n = 3)	Sub District Hospitals (n = 2)	Trust Hospitals* (n = 1)	Medical Colleges* (n = 3)	Other	Total
**Key Informant Interviews**	Hospital Administrator	2	-	1	2	-	**5**
	Pediatrician	4	-	1	3	-	**8**
	Medical Officer	3	2	-	1	-	**6**
	In-Charge Sister	2	-	2	3	-	**7**
	Staff Nurse	-	2	-	-	-	**2**
	Maintenance Contractor	-	-	-	-	2	**2**
	Equipment Manufacturer	-	-	-	-	3	**3**
	Ministry of Health Personnel	-	-	-	-	2	**2**
**Focus Group Discussions**	Residents	-	-	-	7	-	**7**
	Staff Nurses	10	-	5	9	-	**24**
**Total Participants**		**21**	**4**	**9**	**25**	**7**	**66**

*Denotes presence of SNCU.

FGD and KII guides were developed, pilot tested, and revised. FGDs and KIIs were conducted on site at each facility by trained study staff (one interviewer, co-author Cherryl Kolhe, MSc, MPH, LMRF Research Associate) and a notetaker) with expertise in qualitative research methods and analysis. At each site, the newborn care unit director or designate determined which staff were eligible for interviews based on the eligibility criteria noted above. The research team arranged meetings with participants from the hospital staff. Initial meetings between the research staff and the hospital staff were initiated to build rapport and schedule interviews at convenient times. The study goals and procedures were explained to the eligible newborn care unit staff before ensuring their participation. Participants were introduced to the study interviewer as an experienced qualitative researcher in the healthcare field. Participants were informed that there would be no adverse consequences of not participating. Those interested underwent informed consent procedures and if they agreed to participate, they were asked to sign the written study consent form. FGDs and KIIs were conducted in private areas at each facility and audio taped. Participants in the KIIs and FGDs were compensated for their time with a payment of approximately USD10. The interviewer and notetaker conferred after each interview to write a summary of the interview process. Each participant participated once, and no repeat interviews or FGDs were conducted. All transcripts were de-identified for analysis. Eleven KIIs and two FGDs were conducted after the lockdown due to Covid-19.

This study was approved by the Boston University Institutional Review Board (H-38641, the Lata Medical Research Foundation Institutional Review Board (RPC #31), and the Indian Council of Medical Research (2019-7899/F1).

### Data analysis

We used a constructivist Grounded Theory approach [11] to understand SNCU staff perspectives on the barriers and facilitators to optimal newborn care. Interviews were conducted in English or Marathi and audiotaped for transcription. All interviews in Marathi were transcribed and translated verbatim from Marathi to English. For each transcript, Ms. Kolhe, the lead data collector, wrote a precis including her reflections on potential biases when conducting the interviews and focus group discussions. These were reviewed weekly with the data analysis team (one PhD medical anthropologist and two masters level public health research assistants). Each transcript was uploaded into NVivo V.12, (Microsoft Excel [12] a free alternative) and coded using line-by-line coding followed by focused coding to identify major themes emerging from the data, a common approach using Grounded Theory. The data analysis team of three coders read through the initial ten transcripts to develop a draft codebook and to ensure consistency across coders. This team of three coders met regularly with the main data collector, Ms. Kolhe, to review the developing codebook. The codebook was updated and revised after reviewing all transcripts and then used to code all the transcripts. The data analysis team constructed a codebook with parent, child, and grandchild codes. The constant comparative method was used to establish analytical distinction and thus make comparisons throughout the analysis. Themes, patterns, and relationships between themes were identified in the analysis. As themes emerged from the data, we compared them across transcripts. As our guiding framework, we used the socio-ecological model to identify themes, patterns, and relationships between themes regarding barriers and facilitators to providing optimal care for neonates in the SNCUs at the individual, interpersonal, facility, and systems levels. Due to study procedures and the Covid-19 lockdown, transcripts were not shared with participants for corrections and findings were not presented to participants for feedback.

### Inclusivity in global research

Our India and US-based team have had a formal partnership for over 25 years that has resulted in over 100 publications and reports focused on maternal and neonatal health. Our team includes researchers from India and the USA. Five of the co-authors on this manuscript are citizens of India, one of whom, Dr. Archana Patel, is a study principal investigator. Additional information regarding the ethical, cultural, and scientific considerations specific to inclusivity in global research is included in the Supporting Information (S1 Text).

## Results

Data were collected during the months leading up to the pandemic and continued through November of 2020. Although this delayed the data collection process, we were able to document barriers specific to Covid-19 as described by the participants.

**Fig 1:**
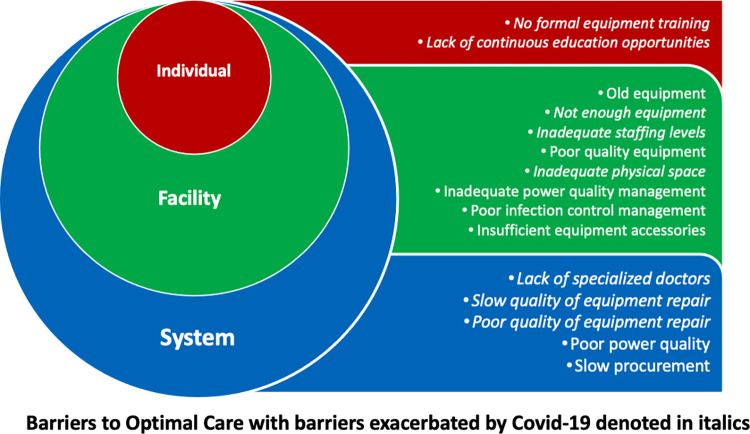
Barriers to optimal care for babies in newborn care units that highlight the impact of Covid-19.

The participants in our study discussed several barriers to providing optimal care for babies in these newborn care units. These barriers can be seen at all levels of the socio-ecological model and are displayed in Fig 1.

### Major barriers to providing optimal care for babies in the newborn care units during Covid-19

The following are the major themes highlighting the barriers identified in the KIIs interviews and FGDs with clinical staff and administrators working in the nine facilities. Covid-19 negatively affected the ability of the units to provide optimal care to neonates in the following ways:

- As a result of Covid-19, the units were even more short-staffed than usual, with trained pediatric nurses and essential equipment diverted from newborn care to attend to patients with Covid-19. The mandatory Covid-19 quarantine also added to the existing duration of absence of essential neonatal staff away from work. The unaccounted quarantine time added pressure on adjusting neonatal duty times for essential staff.
- Regular training of neonatal nursing staff was also disrupted due to Covid-19, leaving many staff without the skills to provide optimal care to neonates. The nurses replacing neonatal staff were not trained in specialized pediatric care. As a result, the training of new staff in neonatal care and equipment became a burden.
- Infection control was also a challenge exacerbated by Covid-19. The staff faced the double burden of protecting neonates from contracting Covid-19 and preventing spread of existing infection, which was exacerbated by shortage of sanitizers that were prioritized for the Covid-19 ward.

#### As a result of Covid-19, the units are even more short-staffed than usual, with trained pediatric nurses and essential equipment diverted from newborn care to attend to patients with Covid-19

Before the Covid-19 pandemic, inadequate staffing of nurses and pediatricians left existing staff exhausted and unable to provide optimal care for large numbers of babies. Low levels of appropriate staffing prevented many facilities from providing optimal care to babies. This situation became critical during Covid-19 when many staff were diverted to Covid-19 and other wards. (Please note that “I” refers to interviewer and “P” refers to participant in the quotes provided throughout).

*I: Ok, what is your opinion on your staff allotment? P: (Long Pause) What can we answer for this? P: We can say we have sufficient staff. We had but because we have made COVID Unit, so some of our staff is posted there. So, the number from here has reduced. I: But other than COVID? P: It was sufficient, and we were subtracted from that. P: First it was sufficient, we could manage, but because of COVID, some staff was taken from each department and posted there, so now staff is a little insufficient.—Staff Nurse*

*I: Sir, do you think shift changes affect baby care? P: Sometimes. Because what happens when a sister is not trained in NICU care comes for this duty, then at that time baby care will be neglected. Because in this COVID-19 era, many sisters are posted in COVID-19 ward. We are getting sisters that are not trained. So, at that time the baby’s’ care may be compromised. Especially those who do not understand the terminologies, how to take care of the baby, how to prevent hypothermia, how to feed the baby, how to give injections to the baby. All these are some things which sisters are not handling properly. I: So, the sisters who are coming instead of sisters for COVID-19 duties, so you are saying they are pediatric sisters and not trained? P: No, they are not pediatric nurses. They are relieving sisters. They are called relieving sisters and not specifically pediatrics. At that time there were some problems. I: So, how do you deal with that? P: Our residents have to be very good. I: Ok. P: Yes, Because the residents are available 24X7. I: But then what if your resident is on COVID-19 duty? P: So, we have to manage with whatever residents are there. Like, if I have 5 residents and 2 are on COVID-19 duty, then 3 will manage. -Pediatrician*

One pediatrician and an in-charge nurse said that baby care may be compromised because trained pediatric nurses are pulled away to staff the Covid-19 wards.

*P: Staffing is less. The ideal ratio is 1:2–3. But because the staff is less, even because of COVID-19 rotation, all the staff is scattered and the staff is disturbed and is less. At NICU we have all technical work. We need trained staff. We can’t just put an IV and leave it at that. Because of COVID-19, our pattern has been disturbed.—In-Charge Nurse*

This pediatrician mentioned his experience serving in the Covid-19 ward and subsequent quarantine, which led to the need to bring in other physicians in his absence.

*P: I was posted on COVID-19 duty on two occasions. I: How long was that for? P: First was for 16 days followed by 7 days of quarantine. Second duty was for 7 days followed by 3 days quarantine. So, in my absence some other doctor was taking care.—Pediatrician*

#### Covid-19 has forced the neonatal care units to divert equipment to other wards

Shortage of equipment leads to sharing of equipment (for example two babies to a warmer) and prioritizing access to limited equipment supply based on severity of illness. For example, staff talked of moving babies who still need the CPAP machine, but are less sick, off the CPAP to enable staff to use it for sicker babies. Providers also discussed how sharing equipment can lead to infections.

*I: Do you share equipment with other units? P: Because of COVID-19, we had to loan it. If there was a positive baby, we loaned some things from our end. I: What did you have to give? P: Cradle, mattress, etc. Photo Therapy, ventilator. CPAP. We had to give. I: Anything else other than that? Like for Labor Room or PICU? P: (Overlapping Responses). Yes, we gave 15 cradles with mattress for COVID-19 and NICU ward.—In-Charge Nurse*

#### Regular training of neonatal nursing staff was also disrupted due to Covid-19, leaving many staff without the skills to provide optimate care to neonates

Training of staff occurs on a routine schedule; however, because of high staff turnover, not all newly hired staff are trained on the proper use of neonatal equipment. If a staff nurse is hired immediately after a training, that nurse may not be trained in months. Lack of training is particularly a problem in the government hospitals. An additional challenge is that Covid-19 interrupted the regular training of staff in the use of the equipment.

*So training is the main challenge here. Training should be focused on the end user. P:-Yes. We are trying hard. But covid–after Covid we will have another training session say after two or three months.—Annual Maintenance Contractor*

*I: Have you been trained for equipment use? P: Nothing special. Based on a specific equipment. But based on our experience and what our seniors have taught, we have taught ourselves. So, we learn on the demo of the new equipment. And in case suppose there is a ventilator, the doctors use it. Sometimes there is a problem and the doctors also don’t know then the biomedical engineer takes our classes. I: So how often does it happen? P: It has stopped since COVID, but otherwise, once in 2–3 months.—In-charge Nurse*

#### Infection control is a challenge, especially during Covid-19

In some facilities, because the units are understaffed, nurses struggle to ensure that all the equipment is kept clean. While cleaning between each baby is the standard, some equipment is only cleaned on a weekly basis. Some units cannot keep the babies for fear of infection and instead stabilize and refer the baby to a higher-level facility. After the Covid-19 lockdown, one participant indicated that hand sanitizers from the SNCU were also diverted to the Covid-19 wards.

*I: How often is equipment cleaned? P: I think we are deficient in that area. Ideally, it should be cleaned daily but we are able to clean it once a week.—Medical Officer*

*I: What about infection control management? P: It is difficult. So, we just stabilize the child and quickly refer. We can’t do more than that. We avoid keeping babies here because we can’t control infection. So, manpower is our primary concern, then infection control is secondary for us.—Medical Officer*

*Because of COVID-19, we are getting fewer sterilizers, but we are diluting it and using as half proportion. But we keep it next to every baby. We do regular dusting.—In-Charge Nurse*

## Discussion

Across the globe, health systems have been severely challenged by the Covid-19 pandemic. In India, many efforts have been underway to mitigate the impact of the epidemic on health services. Despite these efforts, hospitals are overrun, staff are burned out, and people with non-Covid-19 related illnesses are not receiving the care they need. Our study was conducted in the months leading up to Covid-19 and continued for eight months after the initial lockdown, albeit at a much smaller scale than before lockdown.

We found several barriers to providing optimal care for newborns, especially exacerbated during the Covid-19 epidemic when trained neonatal staff and equipment were diverted to Covid-19 wards. Pre-Covid-19 barriers to care included lack of training in the use of medical equipment at the individual level; old and poor-quality equipment, staff shortages and consequent burnout, and poorly designed space at the facility level; and slow procurement of equipment, slow or poor-quality equipment repair, and chronic shortages of nursing staff, specialized doctors, and biomedical engineers at the systemic level.

In our study, unit staff were not always trained to use the equipment by the suppliers when they first began working in the neonatal care units. Equipment suppliers usually train staff only once when the equipment is first installed. New hires are trained by colleagues who may not know all the functions of the equipment. Several other studies have found that lack of training in the use of equipment is a key barrier to optimal care for neonates [6,13–16]. In our study, this problem was further exacerbated by Covid-19 when neonatal nursing staff who were well-trained in the use of the equipment were diverted to the Covid-19 wards and replaced by nurses who were not properly trained.

All government facilities in our study suffered from staff shortages, an endemic problem in India [17]. In our study, Trust hospitals appeared to have more resources than government hospitals and can therefore improve their staff-to-patient ratio so that all babies receive the care they need and to prevent staff burnout. However, both government and Trust hospitals have been stretched thin during Covid-19. A recent review by Kavitha et al. found that at the beginning of the pandemic in India when our study took place, there were no clear guidelines regarding the care of pre-term babies, leaving staff overwhelmed, which led to disruptions in routine care [18]. Other studies have found staff shortages and diversion of resources as a result of Covid-19 [9,19]. A recent survey of 1120 neonatal health care providers from 62 countries found that shifting neonatal care staff to other wards is a common complaint during Covid-19 [9,20,21].

Our study revealed that, despite the pandemic, there was no difference in the rates of admissions at the neonatal care units or their mortality rates, which just served to worsen the severity of the crisis. Overcrowding of the neonatal health care units is common in India, resulting in sharing of equipment (e.g., warmers) and creating the circumstances in which infections can spread. A qualitative study assessing barriers and facilitators for optimal obstetric and neonatal emergency care in primary care facilities in Bihar, India, reported three fourths of participants indicated a lack of physical resources is a barrier to providing adequate care for patients [6]. In our study, we found that Covid-19 has only made this situation worse as both equipment and were diverted to other wards. Systemic barriers to optimal neonatal care include the dearth of trained nurses and pediatricians, slow procurement of equipment, poor quality equipment, and slow repair of equipment. Other studies have also documented chronic staffing shortages in India, equipment procurement delays and malfunction as major barriers to neonatal care [6,9,16,17,19].

Our study found that procurement of equipment, especially in government facilities, involves lengthy bureaucratic procedures. Poor quality equipment, frequent breakdowns, and the slow repairs of equipment are common problems in the neonatal units in India [16]. These problems can lead to sharing of equipment and subsequent infant exposure to infection. Covid-19 also delayed equipment repairs and trainings. Our study findings suggest that AMC contractors need to be held accountable for regular maintenance and timely repairs, but also that government facilities need to invest in procuring reliable equipment.

Tertiary care trust hospitals enjoyed more decision-making power chiefly because of the autonomy they possessed. They also tended to have greater funding. In comparison, although the government hospitals bear most of the patient burden of severely ill newborns, they faced hurdles in the bureaucracy that either limited or delayed their funding [22,23]. The medical colleges under this study did not face many staffing shortages compared to government facilities due to the availability of medical and nursing students. In addition, these colleges have specialized pediatricians and are willing to admit high risk neonates [24,25].

This study has several strengths. First, our research team members were highly trained and have several years of experience conducting qualitative research in maternal and child health in the Indian context. Our analytical team systematically coded and analyzed the data using an intensive Grounded Theory approach to identify the major themes, patterns, and relationships between themes that focused on the barriers and facilitators to providing optimal care to neonates, including the challenging environment caused by Covid-19. The data from our study were collected in the pre and post Covid-19 period, providing stark contrasts between neonatal care across this period. Most multi country studies conducted during this time were online and survey based [19]. Our study offered an in-depth insight into the on-the-ground realities by visiting these sites and conducting in person interviews.

Based on the findings of this study, we recommend the following actions to better respond to similar crises in the future.

*Staff Training*: Despite the threat of a pandemic, the unpreparedness for a scenario as this was evident everywhere. Although guidelines for Covid-19 preparedness were instituted, these were not tailor-made for each department, making it challenging for staff from non-Covid-19 wards to implement them in specialized care units, including the neonatal care units. This lack of curated guidelines was also relevant in the international standards of implementation, where local health facilities like the ones in this study, with existing limited resources, failed to keep up. Although hospital administrators and department heads are well versed in the delivery of care, they may benefit from staff management trainings that emphasize staff allocation and patient triage in a manner that always ensures the availability of at least some experienced and trained staff on duty in a situation where staff are diverted [26]. Covid-19 had a major impact on the psychosocial health of staff and care givers. Hence, staff trainings should also emphasize managing anxiety, workload and stress that were the latent effects of the Covid-19 pandemic.

*Equipment Training*: A major challenge during Covid-19 was the shortage of equipment due to sharing and diversion of equipment to Covid-19 wards. While the remaining equipment was left to use for the relieving staff, these staff were unequipped to handle this equipment. The lockdown and travel restrictions prevented them from acquiring hands-on training by equipment manufacturers. Staff had to rely on their peers for equipment handling, which affected patient care. Hospital administrators may consider innovative training models to address the training gap in adverse situations as Covid-19; for example, creating videos on equipment use and conducting online equipment training for staff.

*Triage*: Inability to prioritize staff duties and neonatal care was one of the shortcomings during the pandemic given the extent and impact of the disease. Unit heads need to hone their triaging skills in times of emergencies such as Covid-19 [27]. In these scenarios it is critical to carefully plan staff responsibilities while balancing the number of untrained staff with trained staff on duty. During staff briefings, senior staff could also emphasize the severity of the pandemic and encourage experienced staff to have an empathic approach and coach the relieving staff during the hand-over to engender a mentoring environment.

This study also has limitations. First, the results of this study cannot be generalized to other types of neonatal facilities (e.g., neonatal intensive care units (NICUs) or to facilities beyond our study catchment area in a district of Maharashtra State. Second, since SNCU directors selected eligible staff to participate in interviews and focus group discussions, it is possible that the individuals deemed eligible might not represent the opinions of other staff who were not selected.

## Conclusion

This study highlights the barriers to providing optimal care for neonates in health care facilities in Maharashtra that have become particularly challenging during Covid-19 and remains important because of the fear of being unprepared for a future similar crisis. Staffing and equipment shortages and lack of training on the use of equipment are major barriers to providing care of at-risk neonates, but our study also highlights poor quality equipment, slow equipment repair and bureaucratic systems to procure new equipment. These staff and equipment barriers intensified during the Covid-19 epidemic and affected critical neonatal care. The barriers seen at the individual, facility, and systems levels will remain a challenge even post Covid-19. Managerial and staff trainings, proper triage and approaching the pandemic situation with emotional intelligence and creativity are some of the solutions for low resource clinical settings. Unit heads should also adapt the global pandemic guidelines as closely possible to their current settings.

For more information, please see this website: https://sites.bu.edu/bb4b/.

## Supporting information

S1 Text Inclusivity in global research. (PDF)

S2 Text Consolidated criteria for reporting qualitative studies (COREQ): 32-item checklist. (PDF)
